# Understanding the Complexity of Hyperglycemic Emergencies: Exploring the Influence of the Type and Duration of Diabetes Mellitus and Its Impact on Mortality

**DOI:** 10.7759/cureus.58916

**Published:** 2024-04-24

**Authors:** Yogesh S, Venkatesan S, Jayaraj A T, Karthigeyan T S, Siva Prasath S, Suriya Prakash S, Selva Krishna R, Sandhiya N, Navvin S, Roshan Prasad

**Affiliations:** 1 Internal Medicine, Madras Medical College and Rajiv Gandhi Government General Hospital, Chennai, IND; 2 Internal medicine, Madras Medical College and Rajiv Gandhi Government General Hospital, Chennai, IND; 3 Internal Medicine, Jawaharlal Nehru Medical College, Datta Meghe Institute of Higher Education and Research, Wardha, IND

**Keywords:** hyperosmolar state, metabolic disorder, hyperglycemia, diabetic ketoacidosis, diabetes mellitus

## Abstract

Background

Diabetes mellitus remains a pressing global health issue, characterized by chronic metabolic dysfunction and the potential for life-threatening acute hyperglycemic emergencies. These emergencies, known as diabetic ketoacidosis and hyperosmolar hyperglycemic states, trigger a series of physiological disruptions. This article delves deeply into how the type and duration of diabetes mellitus affect the occurrence of hyperglycemic emergencies and mortality rates.

Methods

The study was conducted at the Institute of Internal Medicine, Rajiv Gandhi General Hospital, affiliated with Madras Medical College, spanning from July 2021 to December 2021. It encompassed both individuals newly diagnosed with diabetic ketoacidosis and patients already undergoing diabetic treatment who developed diabetic ketoacidosis and hyperosmolar hyperglycemic states.

Results

Within the study cohort of 110 patients, 37.27% were diagnosed with Type 1 diabetes mellitus, while 62.73% were classified as Type 2 diabetes mellitus patients. Among these individuals, 23.60% were newly diagnosed with diabetes, 22.70% had been diabetic for less than one year, 47.30% had a diabetic history of two to five years, and 6.40% had been diabetic for over six years. However, upon investigating the relationship between diabetes duration and mortality rate, no statistically significant findings were observed.

Conclusion

Hyperglycemic emergencies represent multifaceted clinical challenges influenced by the interplay of various factors, including the type and duration of the disease. By maintaining effective management of hyperglycemia from the outset and sustaining it throughout their lives, people with diabetes can improve their physical and mental health and reduce the likelihood of developing long-term complications that may negatively impact their overall well-being.

## Introduction

A major threat to global health is diabetes mellitus (DM), a chronic metabolic disorder with high blood glucose levels. As of 2021, approximately 53.5 crore individuals worldwide were reported to be living with diabetes. If current trends continue, this number is expected to rise to 70 crores by the year 2045 [1]. Chronic adversities, including cardiac disease, blindness, renal insufficiency, disarticulation, and cerebrovascular disease, represent significant contributors to disease burden and early death in individuals with diabetes. Alongside symptoms associated with hyperglycemia such as polydipsia, polyuria, and unexplained weight loss, diabetes can also trigger life-threatening hyperglycemic emergencies. These emergencies, namely diabetic ketoacidosis (DKA) and hyperosmolar hyperglycemic state (HHS), are distinguished by severe disruptions in glucose metabolism, precipitating a series of physiological imbalances [2].

The two primary types of diabetes are Type 1 diabetes mellitus (T1DM) and Type 2 diabetes mellitus (T2DM), with each having unique etiologies and pathophysiological mechanisms. T1DM, typically diagnosed during childhood or adolescence, arises from the self-destructive immune response of pancreatic beta cells, resulting in a total absence of insulin production [3]. T2DM is a type of diabetes mellitus that is typically identified in adults. It is characterized by insulin resistance and relative insulin insufficiency, which result from a combination of genetic and lifestyle factors [4].

The fundamental differences between these diabetes types contribute to variations in the onset, progression, and management of hyperglycemic emergencies. T1DM, prevalent in younger individuals, tends to exhibit abrupt and severe manifestations of hyperglycemic emergencies [5]. The absence of endogenous insulin necessitates exogenous insulin therapy, and any interruption in insulin administration can quickly escalate into DKA. In contrast, T2DM, marked by insulin resistance, often manifests with a gradual onset of hyperglycemic emergencies, allowing for a broader window of intervention [6].

Understanding the impact of diabetes mellitus on hyperglycemic emergencies requires delving into the underlying mechanisms. DKA arises from the breakdown of fats, generates ketone compounds, and induces an acidotic state [7]. Inadequate insulin levels in T1DM allow for uncontrolled lipolysis and ketogenesis, contributing to the characteristic acidotic state of DKA. HHS, commonly observed in individuals with T2DM, is characterized by profound hyperglycemia, excessive fluid loss, and impaired consciousness. In T2DM, insulin resistance and poor insulin secretion set off a harmful cycle that leads to high blood sugar, osmotic diuresis, and loss of extracellular fluid, which finally leads to the hyperosmolar state of HHS. The interaction between insulin deficiency and resistance in both types of diabetes significantly contributes to the onset of hyperglycemic emergencies. These metabolic instabilities arise from a mix of increased levels of counterregulatory hormones such as glucagon, catecholamines, cortisol, and growth hormone and either absolute or relative insulin insufficiency [8].

The duration of diabetes mellitus serves as a critical determinant of the risk and severity of hyperglycemic emergencies. Long-standing uncontrolled diabetes predisposes individuals to progressive beta cell dysfunction, impaired glucagon response, and diminished catecholamine counter-regulation [9].

The current research probes to investigate the influence of both the type and duration of diabetes mellitus on hyperglycemic emergencies, specifically DKA and HHS. The study will examine the relationship between diabetes types (Type 1 and Type 2) and both the severity and frequency of these situations. It will also investigate how long diabetes has existed and how that affects the probability and severity of hyperglycemic episodes, examining individuals with newly diagnosed diabetes as well as those with varying durations of the condition. Furthermore, the study seeks to assess the combined effect of diabetes type and duration on mortality rates in cases of hyperglycemic emergencies, providing insights into the relationship between these factors and patient outcomes.

## Materials and methods

Study design and setting

A prospective study carried out at the Institute of Internal Medicine at Madras Medical College, Rajiv Gandhi General Hospital, served as a single-center investigation. The study spanned six months, commencing from July 2021 to December 2021. This cross-sectional study followed the Strengthening the Reporting of Observational Studies in Epidemiology (STROBE) recommendations, which involved careful drafting and article preparation.

Selection criteria

In this cross-sectional study, 110 patients with hyperglycemic situations who were hospitalized in the Intensive Care Unit and Internal Medicine ward at Rajiv Gandhi Government General Hospital, Madras Medical College, the tertiary care center, were included. To improve the analysis, Madras Medical College's Department of Biochemistry and Department of Diabetology worked together on the study. The study commenced with the Institutional Ethics Committee's clearance, obtaining written informed consent from all subjects prior to initiating any testing procedures.

Inclusion

Patients with Type 1 and Type 2 diabetes, including both symptomatic and asymptomatic individuals, were included in the study. It encompassed people who had just received a diabetes diagnosis and those who were receiving therapy for the disease but were either developing hyperosmolar hyperglycemic states or diabetic ketoacidosis.

Exclusion

Participants with additional medical conditions such as renal failure, liver disease, malignancies, or any other conditions potentially influencing glucose metabolism or contributing to hyperglycemic emergencies were also considered in the study.

Pregnant individuals were excluded, recognizing that pregnancy can significantly influence glucose levels and potentially lead to hyperglycemic emergencies unrelated to diabetes type or duration. Participants taking medications known to substantially affect blood glucose levels, such as corticosteroids, specific antipsychotics, or immunosuppressive drugs, were not included in the study. Individuals with incomplete medical records or missing data concerning their diabetes type, duration, or history of hyperglycemic emergencies were excluded from the analysis. Participants who did not provide informed consent to partake in the study were excluded to uphold ethical research practices.

Biochemical investigations

Through a thorough history-taking process, the study elicited presenting symptoms such as nausea, vomiting, mental disorientation, thirst, polyuria, weight loss, weakness, stomach pain, and coma. A clinical examination was then conducted to evaluate the common modes of presentation.

Patients exhibiting symptoms and signs of hyperglycemic emergencies underwent a series of initial biochemical investigations to aid in the diagnosis, including a complete blood count, plasma glucose measurement, assessment of blood and urine ketone compounds, evaluation of serum electrolytes, determination of blood urea and serum creatinine concentrations, arterial blood gas analysis, electrocardiogram assessment, and, if clinically indicated, blood, urine, and throat cultures. Additionally, chest imaging was performed if a pulmonary infection was suspected. Hemoglobin A1c (HbA1c) testing was also conducted to potentially offer insights into the degree of metabolic control.

The Rothera's nitroprusside test, which involves making a reagent with sodium nitroprusside, ammonium sulfate, and anhydrous sodium carbonate, was used to identify urine ketone bodies. The test revealed the existence of ketone bodies based on the color shift that occurred after one minute.

The presence of urine ketone bodies was detected using Rothera’s nitroprusside test. This involved the following steps: First, a solution was prepared by mixing 10,000 milligrams of ammonium sulfate, 300 milligrams of sodium nitroprusside, and 5,000 milligrams of anhydrous sodium carbonate. Next, 500 to 1,000 milligrams of this solution were placed into a test tube, followed by the addition of a drop of fresh urine. After allowing one minute to pass, any change in color was observed. Ketone bodies in the urine underwent a reaction with nitroprusside under alkaline conditions, resulting in the formation of a purple-colored ring.

The biochemical criteria used to diagnose hyperglycemic hyperosmolar state (HHS) include a plasma glucose level exceeding 600 mg/dL, an arterial pH above 7.30 or a venous pH greater than 7.25, serum bicarbonate levels over 15 mg/dL, the presence of small ketonuria or absence of small ketonemia, an effective serum osmolality surpassing 320 mOsm/kg, and manifestations such as obtundation, combativeness, or seizures in around 50% of cases. In contrast, the diagnostic criteria for diabetic ketoacidosis (DKA) involve hyperglycemia of about 200 mg/dL, venous pH below 7.3 or serum bicarbonate below 15 mg/dL, the presence of ketonemia (blood ß-hydroxybutyrate levels equal to or greater than 3 mg/dL), or moderate to large ketonuria. Additionally, the severity of DKA is categorized based on the degree of acidosis: mild if venous pH is below 7.3 or serum bicarbonate is under 15 mg/dL; moderate if pH drops below 7.2 and serum bicarbonate drops below 10 mg/dL; and severe if pH is less than 7.1 and serum bicarbonate is less than 5 mg/dL.

Statistical analysis

The gathered data was inputted into a Microsoft Excel (Microsoft Corporation, Redmond, Washington, United States) spreadsheet, after which we calculated the mean and standard deviation. Analysis of the documented data was conducted utilizing IBM SPSS Statistics for Windows, Version 20 (Released 2011; IBM Corp., Armonk, New York, United States). The correlation between study parameters was examined using Pearson correlation, with the data demonstrating a normal distribution. The correlation coefficient value, indicating either a positive (direct correlation) or negative (inverse correlation) relationship, was analyzed. The significance was established at a value of p less than 0.05.

## Results

The study included 110 individuals who satisfied the selection criteria mentioned above and were classified as T1DM and T2DM, as mentioned in Table 1.

**Table 1:** Type of diabetes and its impact on hyperglycemic emergencies Type 1 DM: Type 1 diabetes mellitus; Type 2 DM: Type 2 diabetes mellitus

Types of diabetes	Frequency	Percentage
Type 1 diabetes	41	37.27%
Type 2 diabetes	69	62.73%
Total	110	100.0%

**Figure 1:**
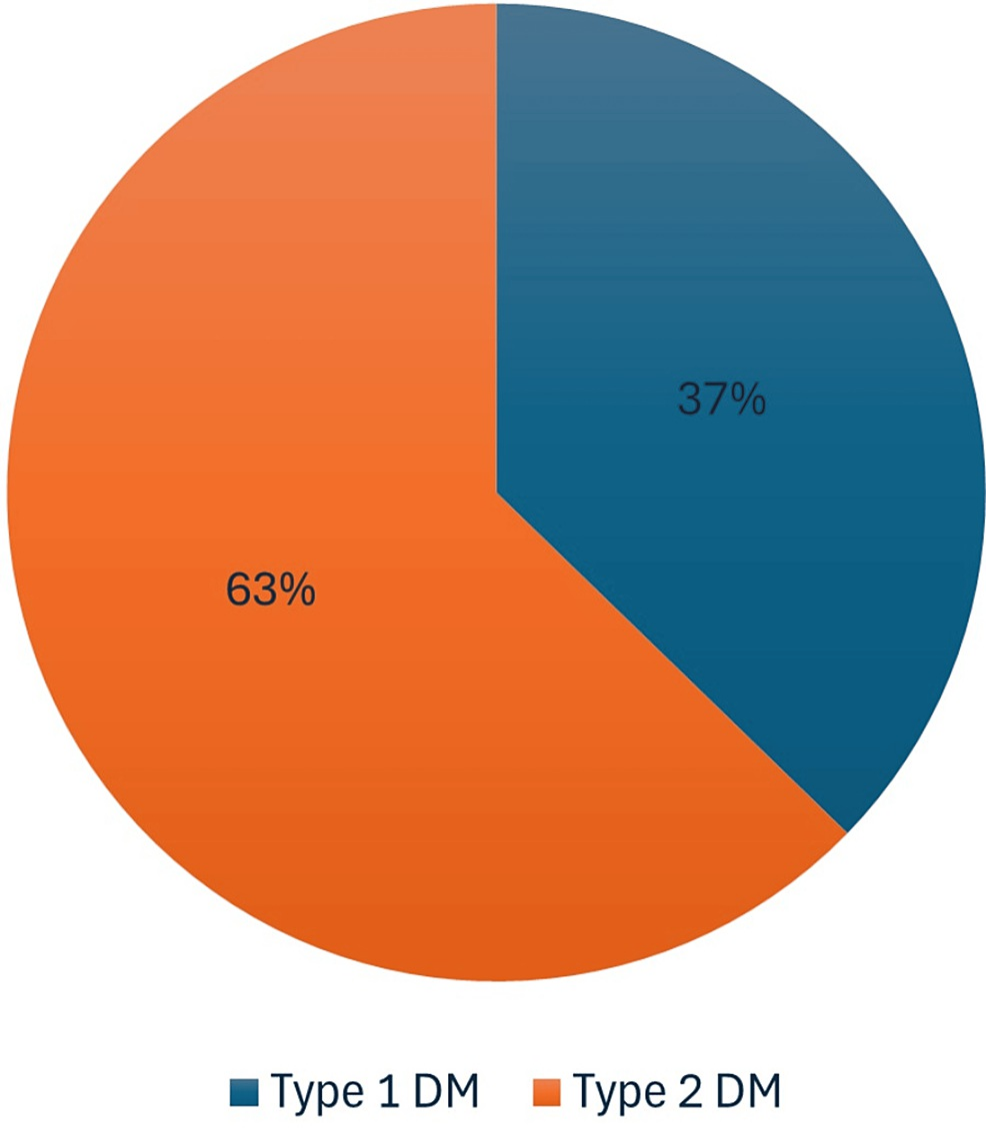
Type of diabetes and its effects on individuals experiencing hyperglycemic emergencies Type 1 DM: Type 1 diabetes mellitus; Type 2 DM: Type 2 diabetes mellitus

Within the study population, 110 patients are present. Figure 1 illustrates that 37.27% were diagnosed with T1DM, while 62.73% were identified as T2DM patients.

In the study population, the diagnosis of diabetes was determined based on the timing and duration of the condition, as in Table 2.

**Table 2:** Duration of diabetes and percentage of hyperglycemic crises DM: diabetes mellitus

Duration of DM	Frequency	Percentage
Newly diagnosed	26	23.60%
<1 year	25	22.70%
2-5 years	52	47.305
>6 years	7	6.40%
Total	110	100%

**Figure 2:**
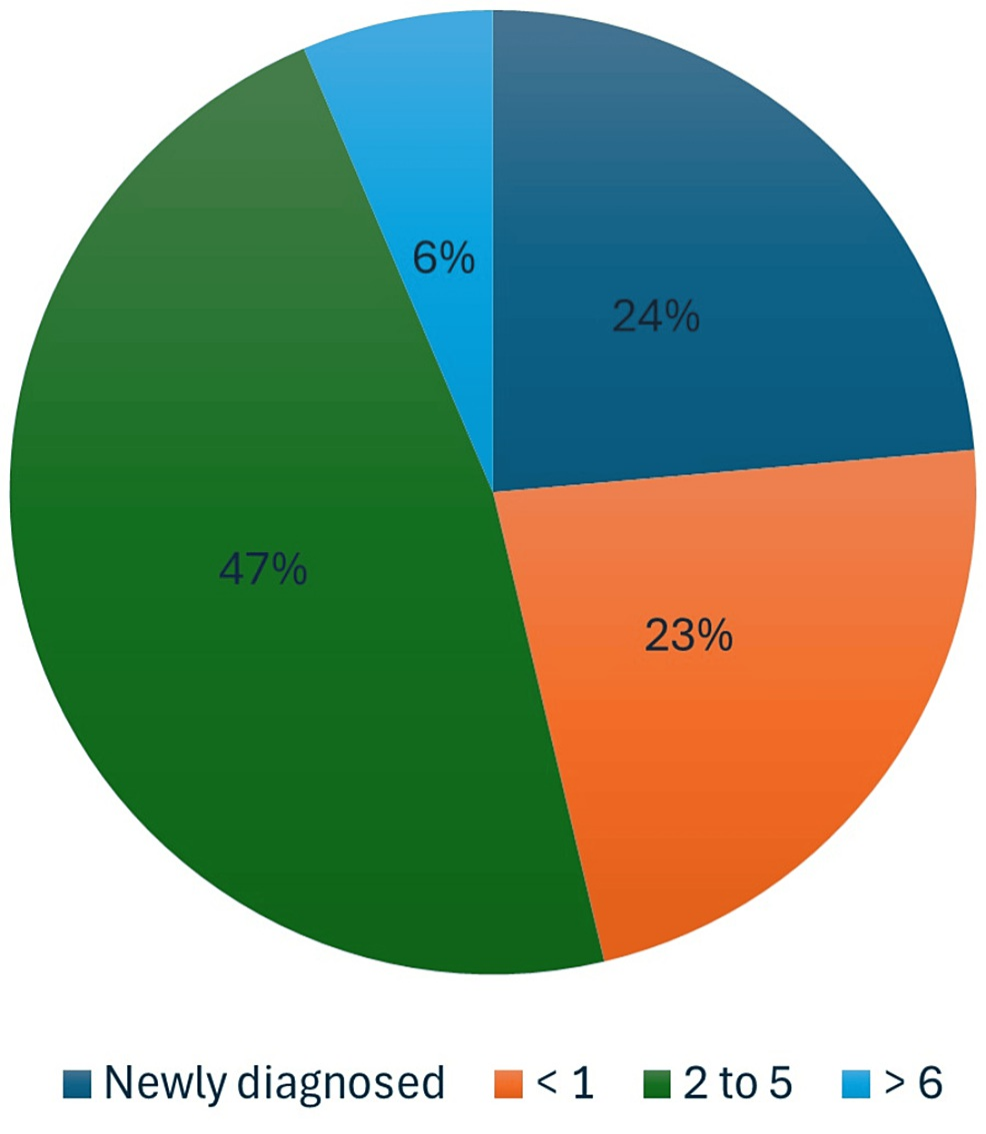
Type of diabetes vs. mortality DM: diabetes mellitus

Based on the type of diabetes, the percentage of mortality is shown in Table 3 and Figure 2. Figure 2 displays the mortality percentage across different durations of the disease.

**Table 3:** Type of diabetes vs. mortality DM: diabetes mellitus

			Mortality		Total	P-value
			No	Yes		
Type of diabetes	Type 1 DM	Count	37	4	41	0.022
		Percentage within Type of DM	90.25%	9.75%	100.0%	
	Type 2 DM	Count	52	17	69	
		Percentage within Type of DM	75.36%	24.64%	100.0%	
Total		Count	89	21	110	
		Percentage within Type of DM	80.91%	19.09%	100.0%	

In this study involving a study population of 110 patients, the impact of diabetes type on mortality was analyzed and depicted in the above table. There is a statistically significant correlation between the type of diabetes and the death rate, as indicated by the computed p-value of 0.022. Of the 41 individuals who received a Type 1 diabetes diagnosis, four (9.75%) died of the condition. On the other hand, 17 individuals (24.64%) out of the 69 patients with Type 2 diabetes died as a result of the condition.

Table 4 illustrates the percentage of mortality aligned with the duration of diabetes.

**Table 4:** Duration of diabetes vs. mortality DM: diabetes mellitus

			Mortality		Total	P-value
			No	Yes		
Duration of DM	Newly diagnosed	Count	18	8	26	0.121
		% within the duration of DM	69.23%	30.77%	100.0%	
	<1	Count	18	7	25	
		% within the duration of DM	72.00%	28.00%	100.0%	
	2-5	Count	44	8	52	
		% within the duration of DM	84.62%	15.38%	100.0%	
	>6	Count	7	0	7	
		% within the duration of DM	100.0%	0.0%	100.0%	
Total		Count	87	23	110	
		% within the duration of DM	79.09%	20.91%	100.0%	

Amidst the study population, from the recently diagnosed diabetes group, eight out of 26 patients (30.77%) succumbed, while for those diagnosed with DM within one year, seven out of 25 patients (28%) experienced mortality. In the two- to five-year history group of DM (52 patients), eight individuals (15.38%) succumbed, whereas in the group with a history of DM exceeding six years, there were no reported mortalities.

This study looked at the relationship between the length of diabetes and how it affects the death rate. In terms of the death rate, the computed p-value of 0.121 indicates no statistical significance.

## Discussion

Two of the most significant warning signs of diabetes mellitus are DKA and HHS, representing opposites in the severity of uncontrolled diabetic states. Persistent diabetes-related complications like neuropathy, nephropathy, and cardiovascular disease increase the seriousness of these emergencies [10].

We conducted a thorough examination of clinical and laboratory data in our study in order to pinpoint variables that might affect a patient's hyperglycemic emergency onset and course. Our findings provide valuable insights into strategies for preventing and detecting these critical events early. Among the 110 patients in our study population, 41 individuals (37.27%) were diagnosed with T1DM, while 69 patients (62.73%) had T2DM. Additionally, within this cohort, 23.60% were newly diagnosed with diabetes, 22.70% had diabetes for less than one year, 47.30% had a diabetes history spanning two to five years, and 6.40% had diabetes for over six years. Notably, the incidence of HHS was more common in older age groups, while the prevalence of DKA was noticeably greater in younger age groups. These observations contribute significantly to our understanding of the demographic patterns associated with these hyperglycemic emergencies.

DKA and HHS share the characteristics of absolute or relative insulin insufficiency and hyperglycemia. Physiologically, these emergencies differ in severity based on factors such as dehydration, ketosis, and metabolic acidosis [11]. Table 5 lists the diagnostic standards for DKA and HHS.

**Table 5:** Diagnostic criteria for DKA and HHS DKA: diabetic keto acidosis; HHS: hyperosmolar hyperglycemic state; *: nitroprusside reaction method; †: effective serum osmolality: 2 [measured Na+ (mEq/l)] + glucose (mg/dl)/18; ‡: anion gap: (Na+) — [(Cl— + HCO — (mEq/l)] Data adapted from ref. [11]

		DKA			HHS
	Mild	Moderate	Severe		Plasma glucose
	(plasma glucose >250 mg/dl)	(plasma glucose >250 mg/dl)	(plasma glucose >250 mg/dl)		>600 mg/dl
Arterial pH	7.25–7.30	7.00 to <7.24	<7.00		>7.30
Serum bicarbonate (mEq/l)	15–18	10 to <15	<10		>18
Urine ketone*	Positive	Positive	Positive		Small
Serum ketone*	Positive	Positive	Positive		Small
Effective serum osmolality†	Variable	Variable	Variable		>320 mOsm/kg
Anion gap‡	>10	>12	>12		Variable
Mental status	Alert	Alert/drowsy	Stupor/coma		Stupor/coma

Inadequate insulin management, illness, the onset of diabetes, and other metabolic stresses are the most common triggering factors for both disorders [12-14]. Diabetes emergencies that pose a serious threat to life are DKA and HHS [15], which are linked to high rates of morbidity [16,17], mortality [18-20], high expenses [2,21], and increased use of medical resources [18-22]. The seriousness of these situations emphasizes how crucial it is to treat diabetes with appropriate management and preventative measures.

Several biochemical abnormalities, such as hyperglycemia, ketonemia, and metabolic acidosis, are indicative of DKA. From 5% to 10% of patients with T1DM experience these episodes. Around 4.6 to eight episodes per 1,000 patients translates to approximately 0.46% to 0.8% of patients with T2DM experiencing DKA [23]. Regarding HHS, there are three main signs: severe hyperglycemia when the plasma glucose over 33 mmol/L corresponds to over 594 mg/dL; hyperosmolarity when serum osmolarity over 320 mOsm/kg remains the same; and mild ketoacidosis when the bicarbonate over 15 mmol/L remains the same. Typically, individuals with T2DM experiencing HHS often present with severe dehydration rather than acidosis. However, in infants with new-onset T1DM, limited access to water may contribute to the development of HHS. In adolescents, the slow development of HHS can be linked to longstanding, unrecognized hyperglycemia, coupled with a high intake of sugar-containing drinks [24]. DKA can also occur in T2DM under conditions of extreme stress, such as serious infection, trauma, or cardiovascular emergencies [25]. Similarly, while HHS predominantly occurs in Type 2 diabetes, it can also be observed in T1DM in conjunction with DKA [26].

In summary, the type of diabetes has a major impact on influencing the risk and characteristics of hyperglycemic emergencies. T2DM, which is more common in adults, is linked with an elevated risk of mortality, particularly when inadequately controlled. In addition to other consequences like kidney disease and retinopathy, this higher mortality risk is often linked to the development of cardiovascular diseases, including heart disease and stroke.

The intricate relationship between diabetes type, associated emergencies, and their consequences highlights the critical importance of effective diabetes management and preventive strategies. The duration of DM is a crucial factor impacting both the risk and severity of hyperglycemic emergencies. In individuals recently diagnosed with diabetes, the likelihood of experiencing hyperglycemic emergencies is generally lower. However, prolonged exposure to elevated blood glucose concentrations causes a progressive decline in pancreatic function, exacerbates insulin resistance, and impairs counter-regulatory responses [27].

These physiological changes compromise the body's ability to effectively handle stressors, making individuals with a prolonged duration of diabetes more susceptible to severe and recurrent hyperglycemic emergencies. Consequently, those with a longer duration of diabetes face an increased risk of experiencing these emergencies, with outcomes potentially further complicated by the presence of comorbidities, thereby making management more challenging. The duration of diabetes mellitus acts as a crucial determinant of the risk and severity of hyperglycemic emergencies. In the early stages of diabetes, the body may retain some residual insulin production, particularly in Type 2 diabetes, which aids in regulating blood sugar levels. However, with time, the pancreas's ability to produce insulin may decline, especially in T2DM, and cells may develop increased resistance to insulin. Both T1DM and T2DM can end up with complications such as DKA and HHS when blood sugar levels persistently remain too high over an extended period. Understanding the influence of diabetes duration on the risk profile offers valuable insights for customizing management strategies and preventive measures in diabetic care.

The cumulative effects of uncontrolled blood sugar levels pose a significant threat, resulting in damage to blood vessels, nerves, and organs. This heightened risk increases the likelihood of diabetic emergencies. As a consequence, it becomes increasingly challenging for individuals to maintain stable blood sugar levels. Diabetic individuals with complications often experience altered symptomatology, delayed treatment-seeking behavior, and an increased susceptibility to infections. Together, these factors contribute to the complexity of managing hyperglycemic emergencies within this population. Effectively addressing and mitigating these challenges is essential for the comprehensive care and well-being of individuals with diabetes.

Limitations

Studies with a limited number of participants may lack statistical power, potentially resulting in less robust and less generalizable findings. Because the study only focuses on the South Indian population, specifically within one city, the extent to which the findings can be extended to other groups, contexts, or conditions may be limited. Applying an alternative statistical method to evaluate a composite score of various indicators could represent a significant advancement in the field.

## Conclusions

Understanding hyperglycemic emergencies in diabetes requires a subtle understanding of their clinical complexities, which depend on factors like illness type and duration. Effective management relies on recognizing distinct presentations in both T1DM and T2DM, particularly in patients with prolonged diabetes who face heightened vulnerability. Proactive monitoring and preventive strategies are paramount, given the documented risks of mortality, hospital readmission, and other severe outcomes. Engagement with diabetes counselors proves pivotal in educating patients on blood glucose management during illness, empowering timely physician intervention, and averting readmissions. Educating patients and families on the early detection and prevention of acute decompensated diabetes is critical. Early and lifelong glycemic control significantly enhances quality of life and mitigates chronic complications, emphasizing the holistic approach to diabetes care through education, observation, and prevention.
